# Perioperative Outcomes in Pregnant Women Who Underwent Surgery for Adnexal Torsion

**DOI:** 10.1055/s-0042-1742403

**Published:** 2022-02-09

**Authors:** Huseyin Ekici, Fırat Okmen, Metehan Imamoglu, Ismet Hortu, Ali Akdemir

**Affiliations:** 1 Department of Obstetrics and Gynecology, School of Medicine, Ege University, Izmir, Turkey; 2 Department of Obstetrics and Gynecology, School of Medicine, Yale University, New Haven, Connecticut, United States; 3 Department of Stem Cell, Institute of Health Sciences, Ege University, Izmir, Turkey; 4 Department of Molecular Pharmacology and Physiology, Morsani College of Medicine, University of South Florida, Tampa, FL, United States

**Keywords:** adnexal torsion, pregnancy, emergent surgery, perinatal outcomes, torção anexial, gravidez, cirurgia emergente, resultados perinatais

## Abstract

**Objective** To evaluate clinical characteristics, maternal and fetal outcomes in pregnant women who underwent surgery for adnexal torsion (AT).

**Methods** All patients, who underwent surgical operation due to AT during pregnancy at the Department of Obstetrics and Gynecology, School of Medicine, Ege University between 2005 and 2020 were retrospectively investigated. Main clinical and perioperative outcomes were evaluated.

**Results** A total of 21 patients who underwent surgery due to AT during pregnancy were included. Of all patients, 61.9% underwent laparoscopy and the remaining 38.1% underwent laparotomy. The most common surgical procedure was adnexal detorsion in both groups (48%). Mean gestational age at the time of diagnosis, duration of surgery and hospitalization were significantly lower in the laparoscopy group, when compared with the laparotomy group ( *p* = 0.006, *p* = 0.001, and *p* = 0.001, respectively.) One of the patients had an infection during the postoperative period. Spontaneous abortion was only observed in one case.

**Conclusion** It can be concluded that the surgical intervention implemented for the exact diagnosis and treatment of AT (laparotomy or laparoscopy) did not have an unfavorable effect on pregnancy outcomes such as abortion, preterm delivery, and fetal anomaly. However, laparoscopy may be superior to laparotomy in terms of advantages.

## Resumo

**Objetivo** Avaliar as características clínicas, e os desfechos maternos e fetais em gestantes submetidas à cirurgia de torção anexial.

**Métodos** Todas as pacientes operadas por torção anexial durante a gravidez no Departamento de Obstetrícia e Ginecologia da Faculdade de Medicina da Universidade de Ege entre 2005 e 2020 foram investigadas retrospectivamente. Os principais resultados clínicos e perioperatórios foram avaliados.

**Resultados** Foram inclusas 21 pacientes operadas por torção anexial durante a gravidez. De todos as pacientes, 61,9% foram submetidas à laparoscopia e as 38,1% restantes foram submetidas à laparotomia. O procedimento cirúrgico mais comum foi apenas a destorção anexial em ambos os grupos (48%). A idade gestacional média no momento do diagnóstico, a duração da operação e da hospitalização foram significativamente menores no grupo de laparoscopia em comparação com o grupo de laparotomia (p = 0,006, p = 0,001 e p = 0,001, respectivamente.) Uma das pacientes teve uma infecção no pós-operatório. Apenas em um caso observamos aborto espontâneo.

**Conclusão** Pode-se concluir que a intervenção cirúrgica implementada para o diagnóstico exato e tratamento da torção anexial (laparotomia ou laparoscopia) não teve efeito desfavorável nos desfechos da gravidez, como aborto, parto prematuro e anomalia fetal. No entanto, a laparoscopia pode ser superior à laparotomia em termos de vantagens.

## Introduction

Ovarian torsion is the complete or partial twisting of the pedicle on its vascular axis, which includes the ovarian arterial and venous vessels, interrupting the blood supply. If the ovarian torsion is accompanied by fallopian torsion, it is called adnexal torsion (AT). The adnexal detorsion (AD) surgery constitutes 2.7% of all gynecological emergent surgeries during pregnancy and may affect women of all ages, particularly during the reproductive period. 1 2 3

The AT is rather rare during pregnancy and its incidence is between 1 and 5 in every 10,000 patients, among cases with spontaneous pregnancy. 4 5 Following the implementation of the assisted reproductive techniques (ART), the number and size of the follicular cysts increase along with the dramatic increase in the risk of torsion. The incidence of torsion may increase in up to 8%, particularly among women with ovarian hyperstimulation syndrome. 6 Although AT is usually encountered in the first trimester, it may also emerge in the second and third trimesters. 7

Early diagnosis is crucial for the preservation of the ovarian and tubal functions and decrease of the related risks of other morbidities. Since there are no exact diagnostic and imaging criteria for the confirmation of the preoperative diagnosis of AT, immediate surgical intervention is also important to preserve ovarian tissue and fertility, and preventing the adverse pregnancy outcomes. Laparoscopy is an effective and safe surgical method mostly preferred for AT treatment in pregnant and non-pregnant women in experienced centers. 8 There are only a limited number of studies focused on the course of the AT and its effects on pregnancy outcomes.

## Methods

All patients, who had undergone surgery due to AT during pregnancy at the Department of Obstetrics and Gynecology, School of Medicine, Ege University (Izmir, Turkey) between 2005 and 2020, were retrospectively investigated. The data related to the demographic characteristics, medical, surgical and obstetric history, findings of the preoperative laboratory and ultrasound examinations, surgery reports, anesthesia, and hospitalization were accessed from the patients' antenatal follow-up files. Pregnancy outcomes such as abortion, gestational age at birth, birth weight, and congenital anomalies were investigated in detail. This study was approved by the Local Ethics Committee of the School of Medicine at Ege University (Approval ID: 20–6.1T/54). Patients whose medical records related to pregnancy monitoring and delivery were not available were excluded from the study.

Regarding imaging methods, we used 2-D ultrasonography, with the Voluson-E8 (General Electric Healthcare, Wauwatosa, WI, USA), 3–9 MHz Transducer scanner and, less frequently, the magnetic resonance imaging (MRI) Magnetom Symphony (Siemens. Erlangen, Germany) 1.5-Tesla scanner. The ultrasound reports were retrospectively scanned, and the findings were divided into three groups: normal ovaries without the presence of cysts or mass, cystic ovaries, and hyperstimulated ovaries. The evaluation of the ultrasound reports showed that the short and long axis of the ovaries were measured in all patients. Furthermore, the mean ovarian diameter was measured, as the calculation of the ovarian volume was not possible. The preoperative white blood counts (WBC) and C-reactive protein (CRP) values were available for all patients. The participating patients were divided into groups according to the preferred surgical intervention (laparoscopy and laparotomy) and the trimester during which AT emerged (first trimester: 5 ^th^ –14 ^th^ gestational weeks; second trimester: 14 ^th^ –28 ^th^ gestational weeks; third trimester: 28 ^th^ gestational week–term). We evaluated the differences for the surgical characteristics, ultrasound findings, and pregnancy outcomes between the two groups.

In patients who went through laparoscopy, the Veress needle was inserted into the umbilicus to create pneumoperitoneum with carbon dioxide gas. In the cases of patients who had previous surgery in the medical history, had suspected periumbilical adhesions, and were in advanced gestational weeks (≥ 15 weeks), the Veress needle was inserted at the Palmer point to create pneumoperitoneum. After the intraabdominal pressure reached 10 to 12 mm Hg, and the 10 to 12 mm primary trocar was placed, the surgeon decided for the placements of the assistant trocars, taking the gestational week and the size of the adnexal mass into consideration. The supine position was selected for first-trimester patients, and left lateral position for the second-trimester patients to avoid aortocaval compression syndrome. In the laparotomy group, the decision of the abdominal incision was made according to the size of the uterus, and size and location of the adnexal mass. In the cases of patients who had undergone cystectomy and salpingo-oophorectomy, all resected materials were referred to the pathological examination. The fetal heartbeat was checked with an ultrasound before and after the intervention. All operations were performed by experienced, high-volume surgeons.

The Statistical Package for the Social Sciences (SPSS, IBM Corp. Armonk, NY, USA) software, version 25.0, was used for the statistical analysis. The normal distribution of the numerical variables was analyzed with the Shapiro-Wilk test (n < 50). The numerical variables were given in mean ± standard deviation (SD), or median (min–max). The categorical variables were given in numbers and percentages. The independent binary sample *t* -test was used in normal distribution, and the Mann-Whitney U test was used in non-normal distribution. The Pearson Chi-square test and the Fisher exact test were used for the categorical variables.

## Results

A total of 21 patients who had undergone surgery due to the AT during pregnancy were retrospectively investigated throughout the study period. The demographic and obstetric characteristics, as well as laboratory findings, were listed in Table 1 . The mean gestational week at the time of diagnosis was found to be 11.9 ± 4.6 (range 6–22). In 14 patients (66.6%) the AT developed in the first trimester, and in 7 patients (33.3%) in the second trimester. Six patients (28.6%) became pregnant after ART implementations (in vitro fertilization: 3 cases; ovulation induction: 3 cases), and the remaining patients became pregnant through spontaneous conception.

**Table 1:** Demographic and clinical characteristics of the patients

Parameter	Results
Maternal age (years)	30 ± 3.9
Parity, n (%)	
Nulliparous	9 (42.8%)
Parous	12 (57.2%)
Surgical history, n (%)	
Laparoscopy	3 (14.3%)
Laparotomy	6 (28.6%)
GA at the time of torsion (weeks)	11.9 ± 4.6
GT at the time of torsion*	
First trimester	14 (66.6%)
Second trimester	7 (33.3%)
Mode of conception, n (%)	
Spontaneous conception	15 (71.4%)
Assisted reproductive technology	6 (28.6%)
Twin pregnancy	1 (4.7%)
Torsion side	
Right	13 (61.9%)
Left	8 (38.1%)
Preoperative WBC (cells/ µL)	12,368 ± 4,864
Preoperative CRP (mg/dL)	1.2 ± 0.8

Abbreviations: CRP, C-reactive protein; GA, gestational age; GT, gestational trimester; WBC, white blood cell. Notes: Data are given as mean ± SD and/or percentage. * No cases were seen in third trimester.

The preoperative imaging examination was mainly performed with ultrasonography and, less frequently, with MRI. We observed cystic lesions in the adnexa (single, multiple or cystic teratoma) in 47.6% of the cases, ovarian enlargement without mass or cyst in 38.1% of the patients, and hyperstimulated ovaries in 14.3% of the patients. The mean ovarian diameter was 77 ± 19 mm (range 52–130). Thirteen patients had their blood flow assessed by Doppler ultrasonography and normal blood flow was observed in 6 patients, who were surgically diagnosed with AT (false-negative rate: 46%). The laboratory analysis showed that both preoperative WBC (12.368 ± 4.864 cells/µL) and CRP (1.2 ± 0.8 mg/dL) were slightly elevated.

**Fig. 1:**
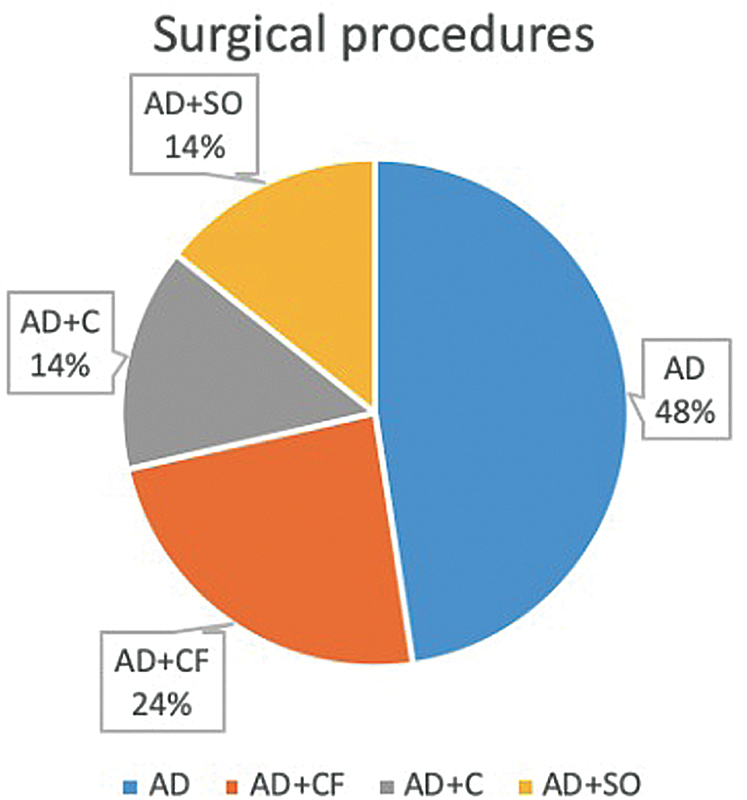
Type of the surgical procedures in adnexal torsion during pregnancy. AD: Adnexal de-torsion only, CF: cyst fenestration, C: cystectomy, SO: salpingo-oophorectomy.

Regardless of the gestational week, laparoscopy and laparotomy were performed in 61.9% (n = 13/21) and 38.1% (n = 8/21) of the patients, respectively. The most common surgical procedures were only adnexal detorsion (48%) ( Fig. 1 ). In one patient, who had AT in her medical history before pregnancy, adnexal fixation was performed to prevent recurrence. Histopathological examination was performed in patients who underwent cystectomy and salpingo-oophorectomy: 2 cases had dermoid cysts; 2 cases serous cysts, one case a paratubal cyst, and 1 case an inflammatory cyst. We encountered no intraoperative complications in any patients and only one second-trimester patient, who underwent laparotomy and salpingo-oophorectomy, developed an infection in the postoperative period. The comparison of the patients according to the implemented surgical method (laparoscopy or laparotomy) showed that there was a statistically significant difference between the groups for the gestational week at the time of diagnosis ( *p* = 0.006), duration of surgery ( *p* = 0.001), and hospitalization ( *p* = 0.001). There was no statistically significant difference between the groups for the gestational week at delivery, birth weight, preterm birth, cesarean section, ultrasound findings, and complications ( Table 2 ).

**Table 2:** Clinical and operative characteristics of the patients

Variable	Laparoscopy group	Laparotomy group	*p* -value
GA at the time of diagnosis (weeks)	9.8 ± 3.4	15.25 ± 4.6	0.006
Operation time (min)	61.6 ± 8.3(45–70)	84 ± 13.4(70–100)	0.001
Duration of hospitalization (days)	3.5 ± 1(2–5)	5.7 ± 1(4–7)	0.001
GA at birth (weeks)	38.1 ± 1.7(34–41)	37.7 ± 2(35–40)	0.549
Birthweight (g)	3,043 ± 338 (2,400–3650)	3,010 ± 292 (2,600–3530)	0.821
Preterm delivery n/N (%)	1/13 (7.7%)	2/8 (25%)	0.271
Cesarean section, n/N (%)	7/13 (53.8%)	4/8 (50%)	0.676
Ultrasonographic findings n/N			
Normal-appearing ovary without cysts	6/13 (46.2%)	2/8 (25%)	0.4
Cystic ovary	5/13 (38.4%)	5/8 (62.5%)	0.387
Hyperstimulated ovary	2/13 (15.4%)	1/8 (12.5%)	0.54
Complications			
Intraoperative n/N (%)	0/13	0/8	NA
Postoperative n/N (%)	0/13	1/8 (12.5%)	0.381

Abbreviation: GA, gestational age; NA, Not applicable. Notes: Data are given as mean ± SD and percentage. Range is given inside the parentheses.

The average gestational age at delivery was 38.1 ± 1.7 weeks (range 34–41 weeks) among women with live birth; three of the cases (14.3%) had delivered before the 37 ^th^ gestational week, and the remaining 18 cases (85.7%) in the 37 ^th^ gestational week or later. The rate of cesarean section was 52.4% (n = 11/21) and all indications depended on the routine fetal, obstetric, or maternal factors. The majority of the patients (95.2%) gave live birth (n = 20/21) and only in one first-trimester patient, who had undergone laparoscopy and AD, spontaneous abortion happened two weeks after surgery. We observed no fetal anomaly in women who had given live birth. The comparison of patients according to the trimester, during which surgery was performed (first trimester and second trimester), showed that there was a statistically significant difference between the groups for the duration of surgery ( *p* = 0.020) and hospitalization ( *p* = 0.009). There was no statistically significant difference between these groups for the abortion rates, gestational age at delivery, birth weight, preterm birth, and cesarean section ( Table 3 ).

**Table 3:** Comparison of surgical and obstetric characteristics at first trimester and second trimester

Variable	First trimester	Second trimester	*p* -value
Operation time (min)	63.4 ± 9.8 (45–80)	84.2 ± 15.1 (70–100)	**0.020**
Hospitalization (days)	3.7 ± 1.3(2–7)	5.5 ± 0.9 (4–7)	**0.009**
GA at delivery (weeks)	38.1 ± 1.8(34–41)	37.7 ± 1.7 (35–40)	0.601
Birthweight (g)	3,018 ± 348 (2,400–3,650)	3,052 ± 259 (2,600–3,420)	0.820
Preterm delivery, n/N (%)	1/14 (7.1%)	2/7 (28.6%)	0.186
Cesarean section, n/N (%)	7/14 (50%)	4/7 (57.1%)	0.761

Abbreviation: GA, gestational age. Notes: Data are given as mean ± SD and percentage. Range is given inside the parentheses.

## Discussion

The AT is one of the most common emergent conditions in obstetrics and gynecology, and it challenges the clinicians because of the maternal and fetal risks. 2 Primarily, AT is suspected because of the nonspecific symptoms such as nausea and vomiting, examination findings (low-grade fever, lateralized lower abdominal pain), and imaging method findings. The definitive diagnosis is done during the surgery. Although it can occur in any trimester, it is more common in the first trimester. 7 In our study, two-third of the cases were in the first trimester. The AT is more common in the first trimester, as the functional ovarian cysts and hyperstimulated ovaries are more common in this trimester. It is relatively rare in the second and third trimesters because these cysts spontaneously regress in these trimesters. Women who underwent ovulation induction and in vitro fertilization are under higher risk of AT. Regarding the studies focused on the effects of AT on pregnancy, 73.2%, 4 48.5%, 9 and 47.9% 10 of the study samples consisted of pregnancies after ART. In our study, this rate was 28.6% and this relatively low rate can be explained by the following factors: implementation of the frozen thaw cycle after the cancellation of the embryo transfer in the same cycle in patients with the hyperstimulated ovary; preference of gonadotropin-releasing hormone antagonists (GnRH antagonists) instead of gonadotropin-releasing hormone analogues (GnRH), which overstimulates the adnexa; early detection of predisposition to ovarian hyperstimulation, and implementation of the appropriate interventions. 11

Transabdominal ultrasonography is frequently the preferred imaging method for AT. Enlarged ovary, solid/cystic/complex ovarian mass, pelvic fluid, and edematous ovarian stroma with peripherally located small follicles are the most common findings in ultrasonographic examinations. 12 The Doppler ultrasound modalities have limited use in AT due to the low sensitivity and operator-dependent usage. 13 If the findings of the ultrasonographic examination are indefinite, MRI may be useful (typically best seen on T2-weighted images). 14 In general, an ovarian diameter equal to or greater than 5 cm is strongly related with AT. 15 16 Hasson et al. 4 reported a mean ovarian diameter of 70 ± 23 mm and a false negativity rate of 61% after the ultrasonographic examination. In our study, the mean ovarian diameter was 77 ± 19 mm and the false negativity rate was 46% in the Doppler ultrasonographic evaluation. Several biochemical parameters such as leukocytosis, CRP, and erythrocyte sedimentation rate were measured in AT cases, and it was found that they were not relevant to diagnosis. 17 18 In our study, we also measured the WBC and CRP parameters and observed slightly elevated levels.

The decision for surgery during pregnancy, particularly in emergencies, is not always easy depending both on the circumstances related to surgery and possible effects of surgery on pregnancy outcomes. In the current literature, it was reported that laparoscopy did not increase the rate of the maternal and fetal complications, and it can be safely and effectively used in the diagnosis and treatment of AT. 19 For this procedure, the optimal gestational week is the second trimester, and several cases who were treated successfully with laparoscopy up to the 34 ^th^ gestational week were reported. 20 In our study, regardless of gestational age, the majority of the cases (61.9%) was treated with the laparoscopic approach. Laparotomy was implemented predominantly between 2005 and 2010 and laparoscopy became more popular with the increase of medical experience with endoscopic surgery. Like in previous studies, the duration of surgery and hospitalization were shorter in cases that underwent laparoscopy. 21 22 Regarding the pregnancy outcomes, no significant difference was observed between laparoscopy and laparotomy. There was no need to convert from laparoscopy to laparotomy in any patient.

Some precautions should be taken to decrease complications during pregnancy related to these procedures, as noted in the literature. These include left lateral recumbent positioning, to minimize compression of vena cava inferior and aorta; initial port placement; and the Veress needle insertion sites should be adjusted according to the gravid's uterine size. Safer alternative sites, such as the Palmer point open technique, can be implemented to prevent devastating complications. Intra-abdominal pressure should not exceed 15 mm Hg during surgery, to minimize pressure-related complications. Moreover, the patient's carbon dioxide levels should be monitored with capnography during surgery. 23 Considering the implemented surgical procedures, AD only was usually sufficient (48%). In cases of patients with cysts, cyst fenestration or cystectomy were preferred. Histopathological examination was performed in 28.6% of the patients, and the dermoid cyst and serous cyst were the most common findings. In their study, Seo et al. 24 performed the pathological examination in 81.8% of the cases, and the most common finding was corpus luteum cyst (42.4%). In principle, only AD or fenestration were performed, particularly in first-trimester patients, to preserve the ovarian reserve, and cystectomy and salpingo-oophorectomy were avoided. In our study, we did not encounter complications in the intraoperative period and only one patient, who underwent laparotomy, developed an infection with no negative effects on the pregnancy outcome.

There are some differences between pregnant women and non-pregnant women in the management of the AT. The choice of anesthesia is generally guided by maternal indications, as well as the site and nature of the planned surgical procedure. However, most abdominal surgical procedures, including laparoscopy, require general anesthesia and muscle relaxation. Preservation of maternal hemodynamic stability, uteroplacental blood flow, and avoidance of maternal and fetal hypoxia throughout surgery, as well as avoidance of preterm delivery, are mandatory. 25 26 General anesthesia is used for the vast majority of laparoscopic, non-obstetric surgeries in pregnancy. Endotracheal intubation with positive pressure ventilation is favored for several reasons: 1. the risk of regurgitation from increased intra-abdominal pressure; 2. the need for controlled ventilation to prevent hypercapnia; 3. the need for relatively high- and peak airway pressures; 4. the need for muscle relaxation (paralysis); and 5. the need for the placement of a nasogastric tube. In addition, when selecting anesthetic drugs, the primary goals are to preserve maternal blood pressure as well as uterine blood flow, and to minimize fetal depression. 27

There are different studies focused on the obstetric alterations in laparoscopy and laparotomy surgeries implemented due to AT in pregnant women. 4 10 28 Oelsner et al. 21 investigated the effects of laparoscopy and laparotomy performed during pregnancy on the obstetric performance and fetal outcomes, and found that the rates of fetal anomalies, abortion, and preterm births were comparable in both groups. Dvash et al. 10 investigated the AT cases managed with laparoscopy during pregnancy and found a high rate of preterm birth. However, they associated this high rate not with laparoscopy but with multiple pregnancies. In our study, the rates of preterm birth and spontaneous abortion were 14.3% and 4.8% respectively, and we observed that the trimester during which surgery was performed in did not change the pregnancy outcomes. Additionally, the type of surgery did not affect pregnancy results either. In women who underwent surgery due to the AT during pregnancy, the timing, mode, and management of delivery were comparable to women with normal pregnancies. The decision for the cesarean section is made on fetal, maternal, and obstetric indications. In our study, the rate of cesarean section was relatively high (52.4%), and we believe this high rate was related to the increasing number of cesarean sections in our country. On the other hand, after the detorsion surgery, postoperative care and instructions following detorsion should include observation for signs of peritonitis or sepsis (fever, worsening abdominal pain, peritoneal irritation signs, hemodynamic instability, etc.), as AT is commonly seen in the first trimester of the pregnancy, and enlarged gravid uterus hinders re-torsion of the ovary during pregnancy in the late weeks. 29

The small sample size and the retrospective nature were the main limitations of the present study. However, as AT during pregnancy is a rare disorder, to conduct a prospective study would be rather difficult. The absence of postnatal data is another limitation of our study.

## Conclusion

If AT is suspected during pregnancy, regardless of the trimester, surgery should not be delayed, to preserve the ovarian and tubal functions and prevent the torsion-related complications. The surgical method (laparotomy or laparoscopy) chosen for the diagnosis and treatment does not have any negative effect on pregnancy outcomes like abortion, preterm birth, and fetal anomaly. In cases with small, simple, non-malignant cystic lesions, adnexal detorsion and cyst fenestration seem a suitable treatment to preserve the ovarian reserve. Furthermore, according to the results of the present study, obstetric outcomes of pregnant women who underwent surgery for AT are generally favorable. If the surgery can be done via laparoscopy in pregnant cases within early gestations, postoperative recovery will be better than open surgery.
